# Learning from the past to develop data analysis curricula for the future

**DOI:** 10.1371/journal.pbio.3001343

**Published:** 2021-07-30

**Authors:** Tracey L. Weissgerber

**Affiliations:** Berlin Institute of Health at Charité—Universitätsmedizin Berlin, QUEST Center, Berlin, Germany

## Abstract

Problems with statistical analyses and the shift towards big data have prompted many researchers to call for improvements in statistics education. This Primer explores a recent study in PLOS Biology that assesses changes in the use of data analysis techniques over time to determine which skills young scientists might need.

## Teaser

Problems with statistical analyses and the shift toward big data have prompted many researchers to call for improvements in statistics education. A recent study in *PLOS Biology* assesses changes in the use of data analysis techniques over time to determine which skills young scientists might need.

Problems with statistical analyses have been well documented in the scientific literature, calling into question many research findings. Common issues include poorly designed or underpowered studies [1], p-hacking [2], pseudoreplication [3], and incorrectly concluding that 2 experimental effects are different without testing for an interaction [4]. Approximately 7% of preclinical stroke and cancer studies excluded animals without explanation, while more than two-thirds of studies did not contain enough information to determine whether animals were excluded [5]. Biased exclusion of animals, particularly in small studies, increases the likelihood of spurious results and overinflated effect sizes [5]. Most physiology papers are missing information needed to determine which type of *t* test or ANOVA was performed and verify the test results [6]. Half of psychology papers that perform null hypothesis significance testing include at least 1 misreported *p*-value, and these errors may alter conclusions in 13% of papers [7]. Statistical training is not always required to complete a PhD in physiology and related fields, and courses may not be designed to meet the needs of students [8]. Findings like these have raised concerns that the statistical training that scientists receive does not sufficiently prepare them to analyze their data. This has prompted many researchers to call for improvements in statistical education and reporting [8,9], especially for scientists working in nonstatistical fields.

The shift toward big data raises additional concerns that the basic techniques that form the backbone of traditional statistics education are woefully inadequate to prepare scientists for the datasets that they will encounter. In a meta-research article published in *PLOS Biology*, Bolt and colleagues seek to identify new data analysis skills that the incoming generation of scientists may need, as big data, machine learning, and other data science approaches are integrated into scientific research [10]. The authors examined time trends in the use of analysis techniques among 1.3 million open-access papers published between 2009 and 2020. Among the study’s many findings, 2 are particularly interesting. First, conventional methods that are traditionally taught in introductory statistics classes, such as *t* tests, ANOVA, linear regression, and other forms of null hypothesis significance testing, remain the most widely used techniques [10]. Although the use of these techniques decreased slightly during the study period [10], these techniques still form the backbone of statistical inference in scientific research. Consequently, addressing misconceptions and training researchers to identify and fix common problems with the use of these techniques should remain a priority for statistical educators. Meta-research articles examining these problems [1–7] are important resources for instructors and students.

A second important finding was that while only a small percentage of papers used advanced data analysis techniques, the use of these techniques is increasing over time [10]. The authors define “advanced techniques” as data analysis skills that are often taught through advanced statistics, data science, or computer science courses. Examples include machine learning, partial least squares discriminant analysis, and regression subset selection. Furthermore, researchers in different fields use different combinations of techniques. The authors list frequently used analysis techniques for each field and propose that this information may be valuable in setting field-specific educational priorities [10]. While this is a laudable goal, we should proceed cautiously. Mentioning a technique in an article is a reasonable proxy measure of use. The observation that many scientists use a technique, however, does not mean that the technique is the most appropriate one to address the research question or that the technique has been used correctly. The fact that conventional techniques are cornerstones in introductory statistics courses may be one reason why these tests are used so frequently. Many scientists rely on the techniques that they know because they don’t have access to a statistician. Furthermore, scientists may not have enough training in data science to identify techniques that are most relevant to their field.

This paper prompts us to consider what additional information one might need to develop modern data analysis curriculum that serves the needs of a new generation of scientists, while avoiding the pitfalls of conventional statistics training. Information on field-specific use of various techniques, as provided by Bolt and colleagues, is one piece of the puzzle. Meta-research, or science of science studies, that identify common problems with the use and interpretation of analysis techniques is also valuable [11]—these data provide a foundation for targeted education programs and interventions to improve data analysis and statistical reporting. While there are many meta-research studies on introductory statistical techniques, science of science studies on the use of newly adopted techniques are urgently needed. This will allow educators and journals to intervene early, preventing the propagation of problematic practices.

As we offer data science training to scientists in different fields, it’s important to learn from our past experience with statistics education. Data scientists should collaborate with experts in specific fields to develop curriculum that meets students’ needs. Statistics courses are often “outsourced” to statistics, public health, or epidemiology departments [8], as many biomedical and biological science departments lack qualified instructors. This approach is likely to be popular for data science training as well. Instructors may be unaware that students from other departments are enrolled in their course. Instructors may also be unfamiliar with common study designs, misconceptions, and poor practices that are widespread in their students’ research fields. Collaboration between data scientists and scientists with field-specific expertise is needed to ensure that course curriculum prepares students to analyze data in their field. Offering field-specific curriculum may be particularly difficult when many departments outsource data science training to a single instructor. Possible solutions include offering shared data science courses for departments with overlapping needs and combining core curriculum with elective modules where students learn techniques that are relevant to their field.

Data science and statistics are complex fields, and the scientific community must be realistic about the level of proficiency that students can achieve while also mastering skills for their own field. When learning new skills, scientists must also learn to recognize their limits. Instructors should train students to determine when a particular technique is appropriate and recognize situations when experts should be consulted. Students should know when complex techniques are needed, even if they can’t implement those techniques themselves. Workshops that teach students how to collaborate with statisticians and data scientists are invaluable. Critical skills include understanding basic vocabulary, knowing how to prepare for a consultation, and collaborating ethically with statisticians and data analysts. Training the next generation of scientists is both an opportunity and a responsibility. The Bolt and colleagues article [10] reminds us that the higher learning community must continuously monitor emerging skills and adapt curriculum to ensure that students receive the training needed for future employment (https://www.weforum.org/agenda/2020/01/how-can-higher-education-adapt-to-a-constantly-evolving-future-of-work and https://www.weforum.org/agenda/2017/07/skill-reskill-prepare-for-future-of-work). Learning from our past experiences with statistics education may help us to prepare young researchers to become the data analysts of the future.
